# Comparing the efficacy of 3D-printing-assisted surgery with traditional surgical treatment of fracture: an umbrella review

**DOI:** 10.1186/s10195-025-00819-0

**Published:** 2025-01-22

**Authors:** Lin Xiao, Peiyuan Tang, Shengwu Yang, Jingyue Su, Wenbo Ma, Han Tan, Ying Zhu, Wenfeng Xiao, Ting Wen, Yusheng Li, Shuguang Liu, Zhenhan Deng

**Affiliations:** 1 Deparment of Orthopedics, Xiangya Hospital, Central South University, Changsha, Hunan China; 2 Xiangya School of Medicine, Central South University, Changsha, China; 3 National Clinical Research Center for Geriatric Disorders, Xiangya Hospital, Central South University, Changsha, China; 4 Department of Orthopedics, The First Affiliated Hospital of Wenzhou Medical University, Wenzhou, China; 5 Geriatrics Center, The First Affiliated Hospital of Wenzhou Medical University, Wenzhou, China; 6 Department of Joint Surgery, Honghui Hospital, Xi’an Jiaotong University, Xi’an, Shaanxi China

**Keywords:** Fracture, 3D printing, Traditional fracture surgery, Umbrella review

## Abstract

**Background:**

The objective of this review is to evaluate the methodological quality of meta-analyses and observe the consistency of the evidence they generated to provide comprehensive and reliable evidence for the clinical use of three-dimensional (3D) printing in surgical treatment of fracture.

**Methods:**

We searched three databases (PubMed, Embase, and Web of Science) up until August 2024. The Preferred Reporting Items for Systematic Reviews and Meta-Analyses (PRISMA) standards were adhered to in this review. The Measurement Tool to Assess Systematic Reviews (AMSTAR) 2 was used to rate the quality and reliability of the meta-analyses (MAs), and Grading of Recommendations Assessment, Development, and Evaluation (GRADE) was used to grade the outcomes. Furthermore, Graphical Representation of Overlap for Overviews (GROOVE) was employed to examine overlap, and the resulting evidence was categorized into four groups according to established criteria for evidence classification.

**Results:**

Results from 14 meta-analyses were combined. AMSTAR 2 gave six meta-analyses a high rating, six MAs a moderate rating, and two MAs a low rating. Three-dimensional printing shows promising results in fracture surgical treatment, significantly reducing operation time and loss of blood for tibial plateau fracture. For acetabular fracture, apart from the positive effects on operation time (ratio of mean (ROM) = 0.74, 95% confidence interval (CI), 0.66–0.83, *I*^2^ = 93%) and blood loss (ROM = 0.71, 95% CI 0.63–0.81, *I*^2^ = 71%), 3D printing helps reduce postoperative complications (odds ratio (OR) = 0.42, 95% CI, 0.22–0.78, *I*^2^ = 9%). For proximal humerus fracture, 3D printing helps shorten operation time (weighted mean difference (WMD) = −19.49; 95% CI −26.95 to −12.03; *p* < 0.05; *I*^2^ = 91%), reduce blood loss (WMD = −46.49; 95% CI -76.01 to −16.97; *p* < 0.05; *I*^2^ = 98%), and get higher Neer score that includes evaluation of pain, function, range of motion, and anatomical positioning (WMD = 9.57; 95% CI 8.11 to 11.04; *p* < 0.05; *I*^2^ = 64%). Additionally, positive results are also indicated for other fractures, especially for operation time, blood loss, and postoperative complications.

**Conclusions:**

Compared with traditional fracture surgical treatment, 3D-printing-assisted surgery has significant advantages and great effectiveness in terms of operation time, loss of blood, and postoperative complications in the treatment of many different types of fractures, with less harm to patients.

**Supplementary Information:**

The online version contains supplementary material available at 10.1186/s10195-025-00819-0.

## Introduction

In the field of orthopedics, fractures rank among the most frequent kinds of injury. The prevalence of fracture is rising globally to become an unavoidable societal health issue owing to the aging of the population, the frequency of transportation accidents, and industrial injuries. Prompt and efficient treatment of fractures is therefore extremely important. To create treatment plans for open reduction and internal fixation (ORIF), plate and screw fixation, and other techniques, traditional surgical treatment of fractures frequently depends on X-ray, computed tomography (CT), magnetic resonance imaging (MRI), and other imaging modalities. These techniques are used to improve fracture healing through precise reduction and stable fixation [1–4]. However, although traditional surgery can meet such treatment needs to a certain extent, the two-dimensional imaging technology that it relies on fails to provide a full understanding of fracture morphology, resulting in poor preoperative planning for some complex fractures, postoperative complications, and unnecessary tissue damage [5]. All of these indicate that the outcome of such management is still far from satisfactory. It is thus meaningful to improve the traditional surgical treatment of fracture to achieve the desired therapeutic effect.

With the rapid development of science and technology, 3D printing technology, as a cutting-edge manufacturing technology, is gradually penetrating into every corner of the medical field and has shown great potential and value especially in fracture treatment. Three-dimensional printing has many applications in surgery, from preoperative planning to the manufacture of personalized implants or auxiliary surgical tools [6, 7]. Three-dimensional printing technology can produce a well-simulated 3D model of the fracture site on the basis of two-dimensional data such as CT or MRI images of the patient [8, 9]. These models not only help doctors to more intuitively understand the shape, displacement, and fragmentation of the fracture, but also can enable surgical simulations, optimize the surgical plan, reduce the risk associated with surgery, and reduce unnecessary tissue damage [10, 11]. Three-dimensional printing is also a potent method in terms of realizing accurate and individualized surgery [12, 13]. It can create personalized implants that perfectly match a patient’s bones on the basis of their bone shape and damage. These implants are not only more biocompatible but also better at promoting fracture healing and bone regeneration [14]. In addition, it can make surgical guides and other tools that match the bone shape of the patient. These tools can precisely guide the entry path and angle of surgical instruments, improving the accuracy and success rate of surgery [15, 16].

Three-dimensional printing is closely related and plays a crucial role in translational medicine and translational orthopedics. In translational medicine, it can quickly transform the results of basic research, such as the development of new printing materials, into clinical applications, helping doctors optimize surgical procedures and improve treatment effects by using patient organ models produced by 3D printing, realizing the transition from “benchside to bedside.” In translational orthopedics, it can assist in precise preoperative planning by means of printed bone models and manufacture orthopedic implants and instruments that are suitable for individuals, transforming the results of scientific research into clinical practice and improving the accuracy of surgeries and the rehabilitation effects of patients [17, 18].

Although the application of 3D printing technology in the treatment of fractures shows many positive effects, the practical clinical application of this technology also has some drawbacks (e.g., elevated cost, not being applicable to all fracture types, etc.). These require us to weigh the advantages and disadvantages in the process of application and try to address the disadvantages.

Increasing interest in 3D-printing-assisted surgery has been accompanied by mounting evidence pointing toward its efficacy in surgery for different kinds of fractures, such as pelvic fracture, proximal humerus fracture, acetabular fracture, etc. Several meta-analyses have shown the effectiveness of different 3D-printing-assisted technology methods for different types of fracture in comparison with traditional non-3D-printing-assisted surgical treatments. Therefore, it is necessary to conduct a comprehensive evaluation of the available evidence to demonstrate and summarize the applications of 3D printing in fracture treatment. The objective of the review is to evaluate the methodological quality of meta-analyses and observe the consistency of the evidence they generated to provide comprehensive, accurate, and reliable evidence for the clinical use of 3D-printing-assisted surgery for fractures. The hypothesis of this study is that 3D printing has great significance for preoperative planning and operation of fracture surgery, yielding significantly better perioperative results.

## Methods

An umbrella review of multiple meta-analyses was carried out. The protocol was registered in the PROSPERO database. A Measurement Tool to Assess Systematic Reviews 2 (AMSTAR 2) was used to gauge the caliber of eligible systematic evaluations, and our review adhered to the Preferred Reporting Items for Systematic Reviews and Meta-Analyses (PRISMA) standards (Supplementary Material A)[19, 20].

### Search methodology

We searched three databases (PubMed, Embase, and Web of Science) up until August 2024. Subject phrases and free words were combined to do the search. “Musculoskeletal Diseases,” “3D printing,” “Printing, Three-Dimensional,” etc. were the main search phrases used (Supplementary Material B).

### Eligibility criteria for studies

The inclusion criteria were: (1) the studies were presented in English language; (2) PRISMA recommendations were followed in the research; (3) in the MAs, traditional surgery served as the control group and 3D-printing-assisted surgery for fractures as the intervention group; (4) sufficient and comprehensive details were provided regarding the outcomes.

The exclusion criteria were: (1) letters, protocols, conference papers, and other nonsystematic reviews and meta-analyses; (2) research of poor quality or that contained a lot of errors; (3) research conducted without human subjects; (4) studies without complete data [21].

### Discovery and processing of overlap

If we found an assessment of the same results in the included meta-analyses (MAs), we had to search for overlap among the original studies to reduce the interference caused by data overlap [22]. This is because the raw data may overlap. This directly provides the total number of both overlapping and non-overlapping preliminary studies and also depicts the overlap graphically. Using GROOVE, we divided the overlap into three groups: low (less than 5%), medium (5% to less than 10%), and high (more than 10%). The GROOVE analysis results are shown in Supplementary Material E.

To address any overlap, we took the following actions: (1) if there was overlap, the conclusions from Cochrane Reviews were given priority over those from non-Cochrane reviews; (2) when multiple non-Cochrane reviews showed substantial overlap, the meta-analysis with the highest AMSTAR 2 score was prioritized. If the AMSTAR 2 scores were equal, the most recently published meta-analyses or those with the most RCTs were included first. In case of either slight or significant overlap, the results of all meta-analyses were applied.

### Extracting data and evaluating quality

Two authors independently gathered, filtered, and evaluated the quality of the methodology and the data. In case of disagreement, the third author assisted in reaching a consensus through shared discussion [23]. From publications that met the eligibility requirements, data on authors, nations, the number of included RCT trials, the number of patients in the 3D-printing-assisted and non-3D-printing-assisted groups, mean age, sex ratio, and outcomes were extracted.

The study focused on several key outcome indicators: operation time (OT), blood loss (BL), postoperative complications (PCs), fracture healing time (FHT), number of fluoroscopies (NOF), and the rate of excellent and good outcome (REGO). AMSTAR 2 was utilized to evaluate the quality and reliability of the included meta-analyses (MAs), while GRADE was employed to assess the level of evidence for each outcome, classifying it as high, medium, low, or very low. In addition, we categorized the resulting evidence into four categories on the basis of evidence classification criteria: I (convincing evidence), II (highly suggestive evidence), III (suggestive evidence), IV (weak evidence), and NS (not significant). For detailed criteria regarding evidence classification, refer to Table 1.

**Table 1:** Evidence classification criteria

Evidence class	Criteria
Class I	> 1000 cases (or > 20,000 participants for continuous outcomes); statistical significance at *p* < 10^−6^ (random effects); no evidence of small study effects and excess significance bias; 95% prediction interval excluded null value; no large heterogeneity (*I*^2^ < 50%)
Class II	> 1000 cases (or > 20,000 participants for continuous outcomes); statistical significance at *p* < 10^−6^ (random effects); largest study with 95% confidence interval excluding null value
Class III	> 1000 cases (or > 20,000 participants for continuous outcomes) and statistical significance at *p* < 0.001
Class IV	Remaining significant associations with *p* < 0.05
Nonsignificant	*p* > 0.05

### Data integration

In terms of data synthesis, we combined findings from systematic reviews using a narrative synthesis approach, and results from meta-analyses were tabulated. Additionally, Supplementary Material F includes a summary table that lists the reviews’ characteristics, revealing that 3D printing technology shows better outcomes across multiple measures for different types of fractures compared with traditional treatments.

## Results

### Search results

**Fig. 1:**
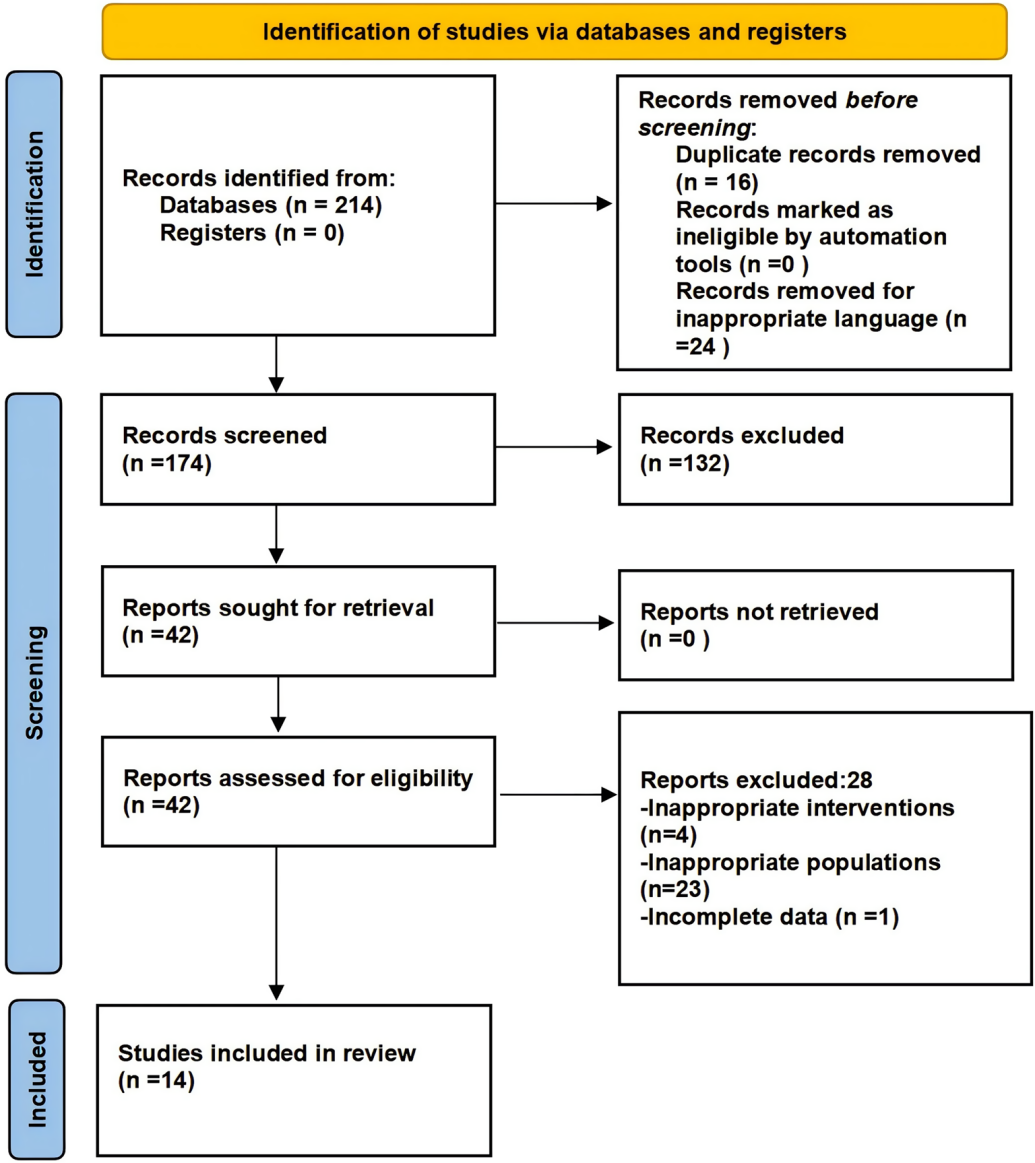
The Preferred Reporting Items for Systematic Reviews and Meta-Analysis (PRISMA) flow diagram to show study selection

A total of 214 studies were identified through searches across three databases. Subsequently, 16 articles were excluded owing to duplication, and 24 articles were excluded owing to inappropriate language. Following a review of abstracts and titles, 132 articles were excluded on the basis of the predefined inclusion and exclusion criteria. Subsequent full-text assessment led to the exclusion of 4 studies owing to inappropriate interventions and 23 studies owing to inappropriate populations suffering other orthopedic disorders but not fractures. Also, we excluded an systematic review because it did not have available data. Ultimately, 14 studies met the inclusion criteria and were included in the analysis. Figure 1 depicts the literature screening procedure.

### Study characteristics

Table 2 lists the basic characteristics of the MAs included in this study. The MAs included in this review were released between 2018 and 2024. This study included the following common fractures: tibial plateau fracture, acetabular fracture, pelvic fracture, and other fractures. A total of 12 studies gave the average age of patients, and in all of these studies, the average age of patients was < 60 years old but > 18 years old (with no minors included). A total of 11 studies gave the sex ratio of included patients, of which only one study had a higher proportion of women than men [3]. A total of 14 studies gave the number of included patients, of which only 1 study had more than 1000 patients [10]. We conducted evidence evaluation on the extracted outcomes (Supplementary Material E). A total of 48 outcomes were rated as level IV, 1 outcome was rated as level III, and 45 outcomes were rated as NS. In addition, we evaluated these outcomes using GRADE, with a total of 48 outcomes rated as moderate, 5 outcomes rated as very low, 27 outcomes rated as low, and 14 outcomes rated as high. We extracted the main conclusions from all the studies included in this review (Supplementary Material F). Six meta-analyses had a high AMSTAR 2 rating [3, 5, 6, 9, 12, 16]. Six meta-analyses had a moderate AMSTAR 2 rating [1, 2, 7, 10, 13, 15]. The rest of the meta-analyses had a low AMSTAR 2 rating [8, 11] (Supplementary Material D).

**Table 2:** Basic information about study patients

Study	Year	Region	Age	Sex ratio (male)	Sample size	Number of studies included	Main outcomes
G. Shi [5]	2021	China	38.15	61%	732	12 (3 RCTs)	OT, BL, PC, NOF, REGO
K. Li [3]	2022	China	59.92	42%	481	9 (4 RCTs)	OT, BL, PC, NOF, FHT, FRR
M. González-Alonso [8]	2021	Spain	NA	NA	744	10	OT, BL, NOF, FHT, PC
Y. He [10]	2022	China	49.74	NA	1165	15 RCTs	OT, BL, NOF, FHT, PC
K. Yammine [2]	2022	America	42.36	60%	673	13 RCTs	OT, BL, NOF, FHT, REGO, FRR
J. Wang [11]	2021	China	42.14	57%	348	7 (5 RCTs)	PC, OT, BL, REGO
L. Wood [6]	2024	United Kingdom	42.46	70%	316	5 RCTs	OT, BL, NOF
J. Bai [7]	2018	China	41.02	NA	486	7 RCTs	OT, BL, REGO, FHT, FRR
L. Xiong [9]	2019	China	NA	51%	521	10(4 RCTs)	OT, BL, NOF, FHT, REGO, FRR, PC
D. Zhu [12]	2020	China	37.33	56%	322	6 (5 RCTs)	OT, BL, NOF, REGO
J. Cao [13]	2021	China	43.61	61%	525	13 (4 RCTs)	OT, BL, FHT, FRR, PC
A. K. X. Lee [16]	2022	Taiwan	39.43	68%	889	17 (3 RCTs)	OT, BL, NOF, FRR, PC
L. Xie [1]	2018	China	44.07	64%	626	15 (10 RCTs)	OT, BL, FHT, PC
D. P. Tu [15]	2021	China	32.89	66%	467	9 (5 RCTs)	OT, BL, NOF, PC

*RCTs* randomized controlled trials, *OT* operation time, *BL* blood loss, *PC* postoperative complications, *REGO* rate of excellent and good outcome, *FHT* fracture healing time, *NOF* number of fluoroscopies, *FRR* fracture reduction rate, *NA* not available

### Results of umbrella review

#### Acetabular fracture

Three MAs [13, 15, 16] reported on acetabular fracture. Regarding how 3D printing helps patients who are suffering acetabular fracture and need surgery, there was overlap between the three MAs. The GROOVE tool was used to identify the overlap of these three MAs, and the specific results are shown in Supplementary Material E. On the basis of the strategies for solving overlapping problems mentioned in the “Methods,” the results of the A. K. X. Lee. et al. study are considered to represent the best available evidence. The findings of the study by A. K. X. Lee. et al. show that, compared with conventional surgical treatment, 3D printing can help shorten operation time (ROM = 0.74, 95% CI 0.66–0.83, *I*^2^ = 93%), lower loss of blood (ROM = 0.71, 95% CI 0.63–0.81, *I*^2^ = 71%), and reduce the occurrence of postoperative complications (OR = 0.42, 95% CI 0.22–0.78, *I*^2^ = 9%). Specific data can be found in Supplementary Material F.

##### Distal radius fracture

Only one study [12] reported on distal radius fracture. The results suggested that 3D-printing-assisted surgery was better than routine surgery in terms of operation time (WMD = −14.52; 95% CI: −21.79 to −7.24; *p* < 0.0001; *I*^2^ = 97%), frequency of intraoperative fluoroscopy (WMD = −2.14; 95% CI −3.43 to −0.85; p = 0.001; *I*^2^ = 95%), and blood loss (WMD = −13.59; 95% CI −18.07 to −9.10; *p* < 0.00001; *I*^2^ = 74%). However, there were no statistically significant distinctions between 3D printing-assisted rehabilitation and traditional rehabilitation in terms of postoperative visual analog scale (VAS) (WMD = −0.55; 95% CI −1.72 to 0.62; p = 0.36; *I*^2^ = 91%) and Gartland–Werley scores (WMD = 0.56; 95% CI −4.18 to 5.30; p = 0.36; *I*^2^ = 0%). Specific data can be found in Supplementary Material F.

##### Proximal humerus fractures

Proximal humerus fracture was reported in only one MA [3]. The study by K. Li et al. reported that 3D-printing-assisted surgery for proximal humerus fractures shortens operation time (WMD = −19.49; 95% CI −26.95 to −12.03; *p* < 0.05; *I*^2^ = 91%), reduces blood loss (WMD = −46.49; 95% CI −76.01 to −16.97; *p* < 0.05; *I*^2^ = 98%), and obtains higher Neer score that includes evaluation of pain, function, range of motion, and anatomical positioning (WMD = 9.57; 95% CI 8.11 to 11.04; *p* < 0.05; *I*^2^ = 64%), with less harm to patients and significant advantages compared with traditional fracture surgery. Specific data can be found in Supplementary Material F.

##### Tibial plateau fracture

Two studies [1, 10] reported tibial plateau fracture. The GROOVE tool was used to identify that there was no overlap between the two MAs, so it was supposed that the data resulting from both studies could be included in the review (Supplementary Material E). As shown in Table 3, both the studies by Y. He et al. and L. Xie et al. indicated that 3D-printing-assisted surgery shortens operation time and reduces blood loss compared with traditional fracture surgery for tibial plateau fracture. Additionally, the study by L. Xie et al. showed the effectiveness of 3D-printing-assisted surgery by comparing the follow-up functional outcomes using Rasmussen score and hospital for special surgery knee score (HSS) score. However, in the study by L. Xie et al., there was no significant difference between 3D-printing-assisted surgery group and non-3D-printing-assisted group in terms of complications and follow-up functional outcomes, although most studies showed higher satisfactory outcome rate with the help of 3D printing technology. Specific data can be found in Supplementary Material F.

**Table 3:** Presentation of outcome data for tibial plateau fracture

Outcome	Author	Type of metric	Effect	95% Cl		*I*^2^ (%)
				Low	High	
Operation time	Y. He[10]	RD	−0.12	−0.16	−0.08	46
	L. Xie[1]	SMD	−2.33	−2.57	−2.09	91.3
Blood loss	Y. He[10]	OR	0.59	0.45	0.77	0
	L. Xie[1]	SMD	−1.51	−1.72	−1.29	88.1

##### Pelvic fracture

Two studies [11, 16] reported on pelvic fracture. The GROOVE tool was used to identify that there was no overlap between the two MAs, so it was supposed that the data resulting from both studies could be included in the review (Supplementary Material E). As shown in Table 3, both the studies by A. K. X. Lee et al. and J. Wang et al. indicated that 3D-printing-assisted surgery reduces operation time, blood loss, and the occurrence of postoperative complications compared with traditional fracture surgery for pelvic fracture. Additionally, the study by J. Wang et al. showed the effectiveness of 3D-printing-assisted surgery by comparing the excellent and good rate of pelvic function and pelvic fractures reduction using Majeed score and Matta score, respectively. Both studies showed that surgery for pelvic fractures performed with the assistance of 3D printing technology resulted in better reduction of fracture, but different methods were used for comparison. Specific data can be found in Supplementary Material F (Table 4).

**Table 4:** Presentation of outcome data for pelvic fracture

Outcome	Author	Type of metric	Effect	95% Cl		*I*^2^ (%)
				Low	High	
Operation time	A. K. X. Lee[16]	ROM	0.74	0.66	0.83	93
	J. Wang[11]	SMD	−2.03	−3	−1.06	92.5
Blood loss	A. K. X. Lee[16]	ROM	0.71	0.63	0.81	71
	J. Wang[11]	SMD	−1.66	−2.69	−0.64	92.3
Postoperative complications	A. K. X. Lee[16]	OR	0.42	0.22	0.78	9
	J. Wang[11]	RR	0.17	0.07	0.44	0

##### Displaced intraarticular calcaneal fracture

Only one study [5] reported on displaced intraarticular calcaneal fracture. The results suggested that 3D-printing-assisted surgery was better than routine surgery in terms of operation time (standardized mean difference (SMD) = −1.86; 95% CI −2.23 to −1.40; *p* < 0.05; *I*^2^ = 83%), postoperative complications (OR = 0.49; 95% CI 0.31 to 0.79; *p* < 0.05; *I*^2^ = 0%), and blood loss (SMD = −1.26; 95% CI −1.82 to −0.69; *p* < 0.05; *I*^2^ = 89%). Specific data can be found in Supplementary Material F.

##### Foot and ankle fracture

Only one study [6] reported on foot and ankle fracture fixation. The results indicated that 3D-printing-assisted surgery was superior to routine surgery in terms of operation time (MD = −23.52; 95% CI −39.31 to −7.74; p = 0.003; *I*^2^ = 99%) and blood loss (MD = 30.59; 95% CI 46.31 to −14.87; p = 0.0001; *I*^2^ = 98%). Specific data can be obtained from Supplementary Material F.

##### Other fractures

Several other studies [2, 7–9] have described other types of fracture, such as appendicular skeleton fracture, pilon fracture, and traumatic fracture. All these studies indicated the positive effects of 3D printing, especially in terms of operation time, blood loss, and postoperative complications. Specific data can be obtained from Supplementary Material F.

## Discussion

This umbrella review synthesizes evidence from multiple meta-analyses, obtaining the main findings that, compared with traditional surgical treatment of fractures, 3D-printing-assisted surgery offers significant advantages in terms of some important outcomes such as operation time, blood loss, and postoperative complications. Such advantage was shown in many different types of fracture, especially acetabular fracture, distal radius fracture, proximal humerus fracture, etc., although some outcomes used to evaluate the efficiency showed no statistically significant distinctions in studies on certain specific types of fracture [10, 15].

When some primary studies compared the 3D printing group with the traditional surgery group, the results for some outcomes show the positive effect of 3D printing with no statistical significance; For example, the study by Shi et al. [5] mentioned that there was no statistically significant difference in postoperative complications and intraoperative X-ray fluoroscopy for 3D-printing-assisted extended lateral approach (ELA) compared with the conventional group for displaced intraarticular calcaneal fracture, which may be related to the small sample size of the trial and the monitoring time of postoperative outcomes. A systematic review by G. Papotto et al. [21] reported that 3D printing has advantages in surgical planning of acetabular fracture in terms of surgical time, reduction of blood loss, quality of fracture reduction, and fixation, but the effectiveness of 3D printing in fracture surgery was demonstrated only for open reduction and internal fixation (ORIF) of fracture treatment. However, this umbrella review reveals the advantages of 3D printing in different surgical methods (such as ELA and ORIF, etc.) for different fractures.

In fracture surgery, 3D printing is mainly used to assist in building solid models to improve visualization and spatial cognition, and to customize personalized fitting instruments. The mechanism of the former is mainly that 3D-printed fracture models convert two-dimensional imaging data into three-dimensional entities. These models can provide intuitive, three-dimensional visual and tactile information to help more accurately determine fracture type, injury degree, and surrounding tissue conditions for good preoperative planning. At the same time, the doctor can also simulate the operation on the solid model to optimize the operative plan [10, 11]; As for the latter, its main advantage is regarding individual patient differences, as 3D printing technology can design and make personalized implants to better match the bone structure of patients, improving the surgical effect and patient comfort [12].

As indicated by many studies, 3D-printing-assisted surgery is more efficient and has greater advantages than traditional surgery for fracture treatment. However, regarding the active use of 3D printing technology in the treatment of related fractures, in addition to considering how to enable the powerful use of this technology, cost effectiveness is also an issue that needs to be paid attention to. Usually, higher-precision 3D printers for medical treatment will cost more than 3D printing equipment for other functions, the operation of 3D printing technology requires professional technicians, and the 3D printing process itself and doctors must spend more time when using 3D-printed models for preoperative planning and simulation. All of this points to the higher cost of 3D-printing-assisted fracture surgery [3]. In terms of effectiveness and benefits, patients can save time on treatment and reduce unexpected additional costs during and after surgery due to complications or inappropriate preoperative planning [15, 16]. Overall, 3D printing for fracture surgery is cost-effective to a certain extent. Although the initial investment of 3D printing for fracture surgery may be high, its significant advantages in reducing trial and error costs, improving surgical success rates, shortening hospital stays, and reducing complications make it significantly cost-effective in the long run. With the continuing maturity of the technology and the further reduction of costs, the application prospects for 3D printing in fracture surgery will broaden [12].

This review has several limitations: (1) Individual meta-analyses have inherent selection, reporting, and publication biases; (2) Many primary studies did not provide detailed information regarding follow-up or specific outcome measurements; (3) The review only included English-language MAs but excluded those published in other languages; (4) Some data from included MAs were not fully available, and comprehensive and unified analysis of the data cannot be carried out; (5) Fractures are not all the same, and so far it is not possible to compare the application of 3D printing for fractures at different sites or at the same anatomical site but with different patterns.

## Conclusions

Compared with traditional fracture surgical treatment, 3D-printing-assisted surgery has significant advantages and great effectiveness in terms of operation time, loss of blood, and postoperative complications in the treatment of many different types of fracture, with less harm to patients.

## Supplementary Information

Additional file 1.

Additional file 2.

Additional file 3.

Additional file 4.

Additional file 5.

Additional file 6.

## Data Availability

Data will be shared upon reasonable request.

## Abbreviations

3D: Three dimensional
MAs: Meta-analyses
RCTs: Randomized controlled trials
