# Supplementary material for: Comparing the efficacy of 3D-printing-assisted surgery with traditional surgical treatment of fracture: an umbrella review
Source: J Orthop Traumatol. 2025 Jan 22;26:3. doi: 10.1186/s10195-025-00819-0 (PMC11754758; doi:10.1186/s10195-025-00819-0)
Supplement: Supplementary file 2 — Additional file 2. [file 10195_2025_819_MOESM2_ESM.docx]

**Embase**

#4 #1 AND #2 AND #3

#3 'systematic reviews as topic'/exp OR 'systematic reviews as topic' OR 'reviews systematic as topic' OR 'systematic review as topic' OR 'umbrella reviews as topic' OR 'meta-analysis as topic'/exp OR 'meta-analysis as topic' OR 'meta analysis as topic'/exp OR 'meta analysis as topic' OR 'clinical trial overviews' OR 'clinical trial overview' OR 'overview, clinical trial' OR 'data pooling' OR 'data poolings' OR 'overviews, clinical trial'

# #2 'printing, three-dimensional'/exp OR 'printing, three-dimensional' OR 'printings, three-dimensional' OR 'printing, three dimensional'/exp OR 'printing, three dimensional' OR 'three-dimensional printings' OR 'three-dimensional printing'/exp OR 'three-dimensional printing' OR 'three dimensional printing'/exp OR 'three dimensional printing' OR '3d printing'/exp OR '3d printing' OR '3d printings' OR 'printing, 3d' OR 'printings, 3d' OR '3-d printing'/exp OR '3-d printing' OR '3 d printing'/exp OR '3 d printing' OR '3-d printings' OR 'printing, 3-d' OR 'printings, 3-d' OR '3-dimensional printing'/exp OR '3-dimensional printing' OR '3 dimensional printing'/exp OR '3 dimensional printing' OR '3-dimensional printings' OR 'printing, 3-dimensional' OR 'printings, 3-dimensional'

#1 'musculoskeletal diseases'/exp OR 'musculoskeletal diseases' OR 'musculoskeletal disease' OR 'orthopedic disorders' OR 'orthopedic disorder'

**WOS**

#1 (((TS=(Musculoskeletal Diseases)) OR TS=(Musculoskeletal Disease)) OR TS=(Orthopedic Disorders)) OR TS=(Orthopedic Disorder) and Preprint Citation Index (Exclude – Database)

#2 (((((((((((((((((((TS=(Printing, Three-Dimensional)) OR TS=(Printings, Three-Dimensional)) OR TS=(Printing, Three Dimensional)) OR TS=(Three-Dimensional Printings)) OR TS=(Three-Dimensional Printing)) OR TS=(Three Dimensional Printing)) OR TS=(3D Printing)) OR TS=(3D Printings)) OR TS=(Printing, 3D)) OR TS=(Printings, 3D)) OR TS=(3-D Printing)) OR TS=(3 D Printing)) OR TS=(3-D Printings)) OR TS=(Printing, 3-D)) OR TS=(Printings, 3-D)) OR TS=(3-Dimensional Printing)) OR TS=(3 Dimensional Printing)) OR TS=(3-Dimensional Printings)) OR TS=(Printing, 3-Dimensional)) OR TS=(Printings, 3-Dimensional) and Preprint Citation Index (Exclude – Database)

#3 (((((((((((TS=(Systematic Reviews as Topic)) OR TS=(Reviews Systematic as Topic)) OR TS=(Systematic Review as Topic)) OR TS=(Umbrella Reviews as Topic)) OR TS=(Meta-Analysis as Topic)) OR TS=(Meta Analysis as Topic)) OR TS=(Clinical Trial Overviews)) OR TS=(Clinical Trial Overview)) OR TS=(Overview, Clinical Trial)) OR TS=(Data Pooling)) OR TS=(Data Poolings)) OR TS=(Overviews, Clinical Trial) and Preprint Citation Index (Exclude – Database)

#4 (((TS=(Systematic Review)) OR TS=(Review,Systematic)) OR TS=(Umbrella Review)) OR TS=(Meta-Analysis) and Preprint Citation Index (Exclude- Database)

#5 #4 OR #3 and Preprint Citation Index (Exclude - Database)

#6 #5 AND #2 AND #1 and Preprint Citation Index (Exclude - Database)

**Pubmed**

(((((((("Meta-Analysis" [Publication Type]) OR ((((((((Meta-Analysis as Topic[Title/Abstract]) OR (Meta Analysis as Topic[Title/Abstract])) OR (Clinical Trial Overviews[Title/Abstract])) OR (Clinical Trial Overview[Title/Abstract])) OR (Overview, Clinical Trial[Title/Abstract])) OR (Data Pooling[Title/Abstract])) OR (Data Poolings[Title/Abstract])) OR (Overviews, Clinical Trial[Title/Abstract]))) OR ("Meta-Analysis as Topic"[Mesh])) OR (((Systematic Review [Publication Type]) OR (Review, Systematic[Title/Abstract])) OR (Umbrella Review[Title/Abstract]))) OR ("Systematic Review" [Publication Type])) OR ((((Systematic Reviews as Topic[Title/Abstract]) OR (Reviews Systematic as Topic[Title/Abstract])) OR (Systematic Review as Topic[Title/Abstract])) OR (Umbrella Reviews as Topic[Title/Abstract]))) OR ("Systematic Reviews as Topic"[Mesh])) AND ((Musculoskeletal Diseases[MeSH Terms]) OR ((((Musculoskeletal Diseases[Title/Abstract]) OR (Musculoskeletal Disease[Title/Abstract])) OR (Orthopedic Disorders[Title/Abstract])) OR (Orthopedic Disorder[Title/Abstract])))) AND ((Printing, Three-Dimensional[MeSH Terms]) OR ((((((((((((((((((((Printing, Three-Dimensional[Title/Abstract]) OR (Printing, Three-Dimensional[Title/Abstract])) OR (Printing, Three Dimensional[Title/Abstract])) OR (Three-Dimensional Printings[Title/Abstract])) OR (Three-Dimensional Printing[Title/Abstract])) OR (Three Dimensional Printing[Title/Abstract])) OR (3D Printing[Title/Abstract])) OR (3D Printings[Title/Abstract])) OR (Printings, 3D[Title/Abstract])) OR (Printing, 3D[Title/Abstract])) OR (3-D Printing[Title/Abstract])) OR (3 D Printing[Title/Abstract])) OR (3-D Printings[Title/Abstract])) OR (Printing, 3-D[Title/Abstract])) OR (Printings, 3-D[Title/Abstract])) OR (3-Dimensional Printing[Title/Abstract])) OR (3 Dimensional Printing[Title/Abstract])) OR (3-Dimensional Printings[Title/Abstract])) OR (Printing, 3-Dimensional[Title/Abstract])) OR (Printings, 3-Dimensional[Title/Abstract])))
