# Supplementary material for: Comparing the efficacy of 3D-printing-assisted surgery with traditional surgical treatment of fracture: an umbrella review
Source: J Orthop Traumatol. 2025 Jan 22;26:3. doi: 10.1186/s10195-025-00819-0 (PMC11754758; doi:10.1186/s10195-025-00819-0)
Supplement: Supplementary file 3 — Additional file 3. [file 10195_2025_819_MOESM3_ESM.docx]

**Supplementary Material C**: Excluded documents and reasons for exclusion

| H. A. O’Connor, 2023[1] | Inappropriate interventions |
| --- | --- |
| V. La Banca, 2024[2] | Inappropriate interventions |
| F. Mancino, 2022[3] | Inappropriate interventions |
| D. A. Schwartz, 2023[4] | Inappropriate interventions |
| Liang, W, 2021[5] | Inappropriate populations |
| Wallace, N, 2020[6] | Inappropriate populations |
| Zhai, W. J, 2024[7] | Inappropriate populations |
| Lu, C, 2023[8] | Inappropriate populations |
| Duan, Y, 2024[9] | Inappropriate populations |
| Hirase, T, 2024[10] | Inappropriate populations |
| Wallace, N, 2020[11] | Inappropriate populations |
| Katiyar, P, 2024[12] | Inappropriate populations |
| Lopez, C. D, 2021[13] | Inappropriate populations |
| Patel, N. A, 2023[14] | Inappropriate populations |
| Oley, M. H, 2024[15] | Inappropriate populations |
| Hassan, M. N, 2019[16] | Inappropriate populations |
| Kumar, S, 2021[17] | Inappropriate populations |
| Pyon, R, 2022[18] | Inappropriate populations |
| Aguado-Maestro, I, 2023[19] | Inappropriate populations |
| Aman, Z. S, 2022[20] | Inappropriate populations |
| Singh, A. K, 2023[21] | Inappropriate populations |
| Maher, D. I, 2022[22] | Inappropriate populations |
| Murray-Douglass, A, 2022[23] | Inappropriate populations |
| Kostakos, T. A, 2021[24] | Inappropriate populations |
| Gernandt, S, 2023[25] | Inappropriate populations |
| Yen, W. W, 2021[26] | Inappropriate populations |
| Anand, S, 2022[27] | Inappropriate populations |
| G. Papotto, 2022[28] | Incomplete data |

Reference:

1. O’Connor, H.A., et al., *3D Printed Orthopaedic External Fixation Devices: A Systematic Review.* 3D Printing in Medicine, 2023. **9**(1).

2. La Banca, V., et al., *Beyond shoulder arthroplasty: Applications of 3D printed patient-specific instrumentation in shoulder and elbow procedures – a systematic review.* Annals of 3D Printed Medicine, 2024. **15**.

3. Mancino, F., et al., *Short-Term Survivorship of 3D-Printed Titanium Metaphyseal Cones in Revision Total Knee Arthroplasty: A Systematic Review.* Orthopedic Reviews, 2022. **14**(4).

4. Schwartz, D.A. and K.A. Schofield, *Utilization of 3D printed orthoses for musculoskeletal conditions of the upper extremity: A systematic review.* Journal of Hand Therapy, 2023. **36**(1): p. 166-178.

5. Liang, W., et al., *3D-printed drill guide template, a promising tool to improve pedicle screw placement accuracy in spinal deformity surgery: A systematic review and meta-analysis.* European Spine Journal, 2021. **30**(5): p. 1173-1183.

6. Wallace, N., et al., *3D-printed Patient-specific Spine Implants: A Systematic Review.* Clinical Spine Surgery, 2020. **33**(10): p. 400-407.

7. Zhai, W.J., et al., *Application of 3D‑printed porous titanium interbody fusion cage vs. polyether ether ketone interbody fusion cage in anterior cervical discectomy and fusion: A systematic review and meta‑analysis update.* Experimental and Therapeutic Medicine, 2024. **28**(1).

8. Lu, C., et al., *Comparison of 3D-printed Navigation Template-assisted Pedicle Screws versus Freehand Screws for Scoliosis in Children and Adolescents: A Systematic Review and Meta-analysis.* Journal of Neurological Surgery, Part A: Central European Neurosurgery, 2023. **84**(2): p. 188-197.

9. Duan, Y., et al., *Comparison of Lumbar Interbody Fusion with 3D-Printed Porous Titanium Cage Versus Polyetheretherketone Cage in Treating Lumbar Degenerative Disease: A Systematic Review and Meta-Analysis.* World Neurosurgery, 2024. **183**: p. 144-156.

10. Hirase, T., et al., *Customized 3-dimensional-printed Vertebral Implants for Spinal Reconstruction After Tumor Resection: A Systematic Review.* Clin Spine Surg, 2024. **37**(1): p. 31-39.

11. Wallace, N., et al., *Three-dimensional Printed Drill Guides Versus Fluoroscopic-guided Freehand Technique for Pedicle Screw Placement: A Systematic Review and Meta-analysis of Radiographic, Operative, and Clinical Outcomes.* Clinical Spine Surgery, 2020. **33**(8): p. 314-322.

12. Katiyar, P., et al., *Three-Dimensional Printing Applications in Pediatric Spinal Surgery: A Systematic Review.* Global Spine Journal, 2024. **14**(2): p. 718-730.

13. Lopez, C.D., et al., *Three-Dimensional Printing for Preoperative Planning and Pedicle Screw Placement in Adult Spinal Deformity: A Systematic Review.* Global Spine Journal, 2021. **11**(6): p. 936-949.

14. Patel, N.A., et al., *Three-Dimensional-Printed Titanium Versus Polyetheretherketone Cages for Lumbar Interbody Fusion: A Systematic Review of Comparative In Vitro, Animal, and Human Studies.* Neurospine, 2023. **20**(2): p. 451-463.

15. Oley, M.H., et al., *Advances in Three-Dimensional Printing for Craniomaxillofacial Trauma Reconstruction: A Systematic Review.* The Journal of craniofacial surgery, 2024.

16. Hassan, M.N., et al., *The bone regeneration capacity of 3D-printed templates in calvarial defect models: A systematic review and meta-analysis.* Acta Biomaterialia, 2019. **91**: p. 1-23.

17. Kumar, S., et al., *Impact of technology in temporomandibular joint reconstruction surgeries: A systematic review.* Journal of Plastic, Reconstructive and Aesthetic Surgery, 2021. **74**(6): p. 1331-1345.

18. Pyon, R., A. Zhang, and A. Lin, *Surgical outcomes of cranioplasty procedures with 3D-printed implants: a systematic review.* Cleft Palate-Craniofacial Journal, 2022. **59**(4 SUPPL): p. 49-50.

19. Aguado-Maestro, I., et al., *CLINICAL APPLICATIONS OF IN-HOSPITAL 3D PRINTING FOR OSTEOPOROTIC TRAUMA PATIENTS: A SYSTEMATIC REVIEW.* Aging Clinical and Experimental Research, 2023. **35**: p. S450.

20. Aman, Z.S., et al., *Improved Accuracy of Coronal Alignment Can Be Attained Using 3D-Printed Patient-Specific Instrumentation for Knee Osteotomies: A Systematic Review of Level III and IV Studies.* Arthroscopy, 2022. **38**(9): p. 2741-2758.

21. Singh, A.K., et al., *Is the Pre-Shaping of an Orbital Implant on a Patient-Specific 3D-Printed Model Advantageous Compared to Conventional Free-Hand Shaping? A Systematic Review and Meta-Analysis.* Journal of Clinical Medicine, 2023. **12**(10).

22. Maher, D.I., et al., *Patient-specific Implants for Orbital Fractures: A Systematic Review.* Ophthalmic Plastic and Reconstructive Surgery, 2022. **38**(5): p. 417-424.

23. Murray-Douglass, A., et al., *Three-dimensional (3D) printing for post-traumatic orbital reconstruction, a systematic review and meta-analysis.* British Journal of Oral and Maxillofacial Surgery, 2022. **60**(9): p. 1176-1183.

24. Kostakos, T.A., et al., *Acetabular reconstruction in oncological surgery: A systematic review and meta-analysis of implant survivorship and patient outcomes.* Surgical Oncology, 2021. **38**.

25. Gernandt, S., et al., *Contribution of 3D printing for the surgical management of jaws cysts and benign tumors: A systematic review of the literature.* Journal of stomatology, oral and maxillofacial surgery, 2023. **124**(4): p. 101433.

26. Yen, W.W., et al., *Current concepts and advances of three-dimensional printing in reconstructive musculoskeletal oncology: A systematic review.* Journal of Long-Term Effects of Medical Implants, 2021. **31**(4): p. 59-71.

27. Anand, S., et al., *Utility of Three-Dimensional Printing for Preoperative Assessment of Children with Extra-Cranial Solid Tumors: A Systematic Review.* Pediatric Reports, 2022. **14**(1): p. 32-39.

28. Papotto, G., et al., *Use of 3D printing and pre-contouring plate in the surgical planning of acetabular fractures: A systematic review.* Orthopaedics and Traumatology: Surgery and Research, 2022. **108**(2).
