# Supplementary material for: Comparing the efficacy of 3D-printing-assisted surgery with traditional surgical treatment of fracture: an umbrella review
Source: J Orthop Traumatol. 2025 Jan 22;26:3. doi: 10.1186/s10195-025-00819-0 (PMC11754758; doi:10.1186/s10195-025-00819-0)
Supplement: Supplementary file 4 — Additional file 4. [file 10195_2025_819_MOESM4_ESM.docx]

**Supplementary material D:** Quality evaluation of included studies**.**

| Study | 1. | 2. | 3. | 4. | 5. | 6. | 7. | 8. | 9. | 10. | 11. | 12. | 13. | 14. | 15. | 16. | | Overall rating |
| --- | --- | --- | --- | --- | --- | --- | --- | --- | --- | --- | --- | --- | --- | --- | --- | --- | --- | --- |
| G. Shi[1] | Y | Y | Y | Y | Y | Y | Y | N | Y | Y | Y | Y | Y | Y | Y | Y | High | |
| K. Li[2] | Y | Y | Y | Y | Y | Y | Y | Y | Y | Y | Y | Y | Y | Y | N | Y | High | |
| M.González-Alonso[3] | Y | Y | Y | Y | Y | Y | Y | N | Y | N | Y | Y | Y | Y | N | Y | Low | |
| Y. He[4] | Y | Y | Y | Y | Y | Y | Y | N | Y | N | Y | Y | Y | Y | Y | Y | Moderate | |
| K. Yammine[5] | Y | Y | Y | Y | Y | Y | Y | Y | Y | N | Y | Y | Y | Y | N | Y | Moderate | |
| J. Wang[6] | Y | Y | Y | Y | Y | Y | Y | Y | Y | N | Y | Y | Y | N | N | Y | Low | |
| L. Wood[7] | Y | Y | Y | Y | Y | Y | Y | Y | Y | N | Y | Y | Y | Y | Y | Y | High | |
| J. Bai[8] | Y | Y | Y | Y | Y | Y | Y | N | Y | Y | Y | Y | Y | Y | N | Y | Moderate | |
| L. Xiong[9] | Y | Y | Y | Y | Y | Y | Y | Y | Y | Y | Y | Y | Y | N | Y | Y | High | |
| D. Zhu[10] | Y | Y | Y | Y | Y | Y | Y | Y | Y | Y | Y | Y | Y | N | Y | Y | High | |
| J. Cao[11] | Y | Y | Y | Y | Y | Y | Y | Y | Y | N | Y | Y | Y | Y | N | Y | Moderate | |
| A. K. X. Lee[12] | Y | Y | Y | Y | Y | Y | Y | Y | Y | Y | Y | Y | Y | N | Y | Y | High | |
| L. Xie[13] | Y | Y | Y | Y | Y | Y | Y | Y | Y | N | Y | Y | Y | Y | N | Y | Moderate | |
| D. P. Tu[14] | Y | Y | Y | Y | Y | Y | Y | Y | Y | N | Y | Y | Y | Y | N | Y | Moderate | |

Scale of item score: N, no; Y, yes.

The AMSTAR criteria are Q1: Did the research questions and inclusion criteria for the review include the components of PICO?, Q2: Did the report of the review contain an explicit statement that the review methods were established prior to the conduct of the review and did the report justify any significant deviations from the protocol?, Q3: Did the review authors explain their selection of the study designs for inclusion in the review?, Q4: Did the review authors use a comprehensive literature search strategy?, Q5: Did the review authors perform study selection in duplicate?, Q6: Did the review authors perform data extraction in duplicate?, Q7: Did the review authors provide a list of excluded studies and justify the exclusions?, Q8: Did the review authors describe the included studies in adequate detail?, Q9: Did the review authors use a satisfactory technique for assessing the risk of bias (RoB) in individual studies that were included in the review?, Q10: Did the review authors report on the sources of funding for the studies included in the review?, Q11: If meta-analysis was performed, did the review authors use appropriate methods for statistical combination of results?, Q12: If meta-analysis was performed, did the review authors assess the potential impact of RoB in individual studies on the results of the meta-analysis or other evidence synthesis?, Q13: Did the review authors account for RoB in primary studies when interpreting/discussing the results of the review?, Q14: Did the review authors provide a satisfactory explanation for, and discussion of, any heterogeneity observed in the results of the review?, Q15: If they performed quantitative synthesis did the review authors carry out an adequate investigation of publication bias (small study bias) and discuss its likely impact on the results of the review?, Q16: Did the review authors report any potential sources of conflict of interest, including any funding they received for conducting the review?

Reference：

1. Shi, G., et al., 3D printing-assisted extended lateral approach for displaced intra-articular calcaneal fractures: a systematic review and meta-analysis. Journal of orthopaedic surgery and research, 2021. 16(1): p. 682.

2. Li, K., et al., 3D printing-assisted surgery for proximal humerus fractures: a systematic review and meta-analysis. European journal of trauma and emergency surgery : official publication of the European Trauma Society, 2022. 48(5): p. 3493-3503.

3. González-Alonso, M., et al., Application of 3D printing in the treatment of appendicular skeleton fractures: Systematic review and meta-analysis. Journal of Orthopaedic Research, 2021. 39(10): p. 2083-2092.

4. He, Y., P. Zhou, and C. He, Clinical efficacy and safety of surgery combined with 3D printing for tibial plateau fractures: systematic review and meta-analysis. Annals of Translational Medicine, 2022. 10(7).

5. Yammine, K., et al., Clinical outcomes of the use of 3D printing models in fracture management: a meta-analysis of randomized studies. European journal of trauma and emergency surgery : official publication of the European Trauma Society, 2022. 48(5): p. 3479-3491.

6. Wang, J., et al., Comparison of the feasibility of 3D printing technology in the treatment of pelvic fractures: a systematic review and meta-analysis of randomized controlled trials and prospective comparative studies. European journal of trauma and emergency surgery : official publication of the European Trauma Society, 2021. 47(6): p. 1699-1712.

7. Wood, L. and Z. Ahmed, Does using 3D printed models for pre-operative planning improve surgical outcomes of foot and ankle fracture fixation? A systematic review and meta-analysis. European journal of trauma and emergency surgery : official publication of the European Trauma Society, 2024. 50(1): p. 21-35.

8. Bai, J., et al., Efficacy and safety of 3D print-assisted surgery for the treatment of pilon fractures: a meta-analysis of randomized controlled trials. Journal of orthopaedic surgery and research, 2018. 13(1): p. 283.

9. Xiong, L., et al., The efficacy of 3D printing-assisted surgery for traumatic fracture: A meta-analysis. Postgraduate Medical Journal, 2019. 95(1126): p. 414-419.

10. Zhu, D., et al., The efficacy of 3D printing-assisted surgery in treating distal radius fractures: Systematic review and meta-analysis. Journal of Comparative Effectiveness Research, 2020. 9(13): p. 919-931.

11. Cao, J., H. Zhu, and C. Gao, A Systematic Review and Meta-Analysis of 3D Printing Technology for the Treatment of Acetabular Fractures. BioMed Research International, 2021. 2021.

12. Lee, A.K.X., et al., Three-Dimensional Printing and Fracture Mapping in Pelvic and Acetabular Fractures: A Systematic Review and Meta-Analysis. Journal of Clinical Medicine, 2022. 11(18).

13. Xie, L., et al., Three-dimensional printing assisted ORIF versus conventional ORIF for tibial plateau fractures: A systematic review and meta-analysis. International Journal of Surgery, 2018. 57: p. 35-44.

14. Tu, D.P., et al., Three-dimensional printing combined with open reduction and internal fixation versus open reduction and internal fixation in the treatment of acetabular fractures: A systematic review and meta-analysis. Chinese Journal of Traumatology - English Edition, 2021. 24(3): p. 159-168.
