# Supplementary material for: Comparing the efficacy of 3D-printing-assisted surgery with traditional surgical treatment of fracture: an umbrella review
Source: J Orthop Traumatol. 2025 Jan 22;26:3. doi: 10.1186/s10195-025-00819-0 (PMC11754758; doi:10.1186/s10195-025-00819-0)

**Supplementary Material E**: Citation matrices for Meta-analyses with overlapping associations

1. **Acetabular fracture**


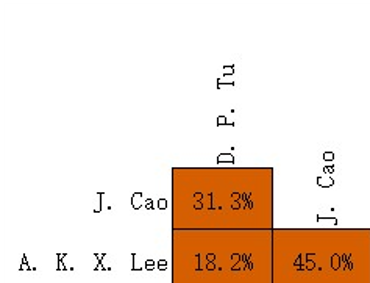


1. **Tibial plateau fracture**


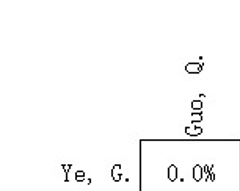


1. **Pelvic fracture**


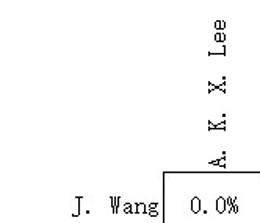

Supplement: Supplementary file 5 — Additional file 5. [file 10195_2025_819_MOESM5_ESM.docx]
