# Supplementary material for: Comparing the efficacy of 3D-printing-assisted surgery with traditional surgical treatment of fracture: an umbrella review
Source: J Orthop Traumatol. 2025 Jan 22;26:3. doi: 10.1186/s10195-025-00819-0 (PMC11754758; doi:10.1186/s10195-025-00819-0)
Supplement: Supplementary file 6 — Additional file 6. [file 10195_2025_819_MOESM6_ESM.docx]

**Supplementary Material F:** Specific d ata of the resultsof the included studies

| Study | Year | Outcome | Type of metric | Effect | 95%Cl | | Effect modle | I2(%) | GRADE | Evidence class |
| --- | --- | --- | --- | --- | --- | --- | --- | --- | --- | --- |
|  |  |  |  |  | low | high |  |  |  |  |
| G. Shi[1] | 2021 | OT | MD | -1.86 | -2.32 | -1.40 | Random | 83 | Low | Ⅳ |
| G. Shi | 2021 | BL | MD | -1.26 | -1.82 | -0.69 | Random | 89 | Low | Ⅳ |
| G. Shi | 2021 | PC | OR | 0.49 | 0.31 | 0.79 | Fixed | 0 | Moderate | NS |
| G. Shi | 2021 | NOF | MD | -0.66 | -1.20 | -0.12 | Random | 76 | Low | Ⅳ |
| G. Shi | 2021 | REGO | OR | 4.09 | 2.03 | 8.22 | Fixed | 0 | Moderate | NS |
| K. Li[2] | 2022 | OT | MD | -19.49 | -26.95 | -12.03 | Random | 91 | Very low | Ⅳ |
| K. Li | 2022 | BL | MD | -46.49 | -76.01 | -16.97 | Random | 98 | Very low | Ⅳ |
| K. Li | 2022 | FHT | MD | -0.95 | -1.78 | -0.12 | Random | 76 | Very low | Ⅳ |
| K. Li | 2022 | NOF | MD | -2.34 | -3.07 | -1.61 | Random | 81 | Very low | Ⅳ |
| K. Li | 2022 | FRR | OR | 6.45 | 1.05 | 39.78 | Fixed | 0 | Moderate | Ⅳ |
| K. Li | 2022 | CS | MD | 7.29 | 5.55 | 9.03 | Fixed | 31 | Moderate | Ⅳ |
| K. Li | 2022 | Neer | MD | 9.57 | 8.11 | 11.04 | Fixed | 64 | Low | Ⅳ |
| K. Li | 2022 | PC | OR | 0.38 | 0.16 | 0.89 | Fixed | 6 | Moderate | Ⅳ |
| K. Li | 2022 | LOH | MD | -1.26 | -4.49 | 1.98 | Random | 99 | Very low | Ⅳ |
| M. González-Alonso[3] | 2021 | OT | SMD | -2.01 | -2.69 | -1.32 | Random | 93 | Low | Ⅳ |
| M. González-Alonso | 2021 | BL | SMD | -1.74 | -2.35 | -1.12 | Random | 90 | Low | Ⅳ |
| M. González-Alonso | 2021 | NOF | SMD | -2.1 | -2.9 | -1.3 | Random | 95 | Low | Ⅳ |
| M. González-Alonso | 2021 | FHT | SMD | -0.15 | -0.49 | 0.19 | Random | 70 | Low | Ⅳ |
| M. González-Alonso | 2021 | PC | RR | 1.28 | 0.72 | 2.25 | Random | 0 | Moderate | Ⅳ |
| Y. He[4] | 2022 | OT | RD | -0.12 | -0.16 | -0.08 | Fixed | 46 | Moderate | Ⅲ |
| Y. He | 2022 | BL | OR | 0.59 | 0.45 | 0.77 | Fixed | 0 | High | Ⅳ |
| Y. He | 2022 | NOF | OR | 0.59 | 0.41 | 0.85 | Fixed | 0 | High | Ⅳ |
| Y. He | 2022 | FHT | OR | 0.46 | 0.33 | 0.63 | Fixed | 0 | High | Ⅳ |
| Y. He | 2022 | RS | OR | 3.08 | 1.89 | 5.02 | Fixed | 0 | High | Ⅳ |
| Y. He | 2022 | HSS | OR | 1.97 | 1.30 | 3.00 | Fixed | 0 | High | Ⅳ |
| Y. He | 2022 | PC | OR | 0.60 | 0.45 | 0.81 | Fixed | 0 | High | Ⅳ |
| K. Yammine[5] | 2022 | OT | SMD | −1.47 | −1.759 | − 1.182 | Random | 60.4 | Low | NS |
| K. Yammine | 2022 | BL | SMD | -1.41 | -1.79 | -1.03 | Random | 77.7 | Low | NS |
| K. Yammine | 2022 | NOF | SMD | -1.25 | -1.87 | -0.64 | Random | 74.3 | Low | NS |
| K. Yammine | 2022 | REGO | OR | 2.05 | 1.12 | 3.85 | Fixed | 0 | Moderate | Ⅳ |
| K. Yammine | 2022 | FRR | OR | 2.64 | 1.15 | 6.05 | Fixed | 0 | Moderate | Ⅳ |
| K. Yammine | 2022 | FHT | SMD | 0.0008 | -0.27 | 0.25 | Fixed | 33.6 | Moderate | Ⅳ |
| J. Wang[6] | 2021 | REGO | RR | 1.29 | 1.15 | 1.44 | Fixed | 0 | High | NS |
| J. Wang | 2021 |  | RR | 1.32 | 1.08 | 1.60 | Fixed | 0 | High | NS |
| J. Wang | 2021 | PC | RR | 0.17 | 0.07 | 0.44 | Fixed | 0 | High | NS |
| J. Wang | 2021 | OT | SMD | -2.03 | -3.00 | -1.06 | Random | 92.5 | Moderate | Ⅳ |
| J. Wang | 2021 | BL | SMD | -1.66 | -2.69 | -0.64 | Random | 92.3 | Moderate | Ⅳ |
| L. Wood[7] | 2024 | OT | MD | -29.52 | -39.31 | -7.74 | Random | 99 | Low | Ⅳ |
| L. Wood | 2024 | BL | MD | -30.59 | -46.31 | -14.87 | Random | 98 | Low | Ⅳ |
| L. Wood | 2024 | NOF | MD | -3.20 | -4.69 | -1.72 | Random | 49 | Moderate | NS |
| L. Wood | 2024 | AOFAS | MD | 2.24 | 0.69 | 3.78 | Random | 0 | Moderate | NS |
| J. Bai[8] | 2018 | OT | MD | -26.16 | -33.19 | -19.14 | Random | 95 | Low | Ⅳ |
| J. Bai | 2018 | BL | MD | -63.91 | -79.55 | -48.27 | Random | 93 | Low | Ⅳ |
| J. Bai | 2018 | PFS | MD | 8.14 | 5.16 | 11.29 | Random | 64 | Low | Ⅳ |
| J. Bai | 2018 | REGO | RR | 1.20 | 1.07 | 1.34 | Fixed | 0 | Moderate | NS |
| J. Bai | 2018 | FRR | RR | 1.35 | 1.19 | 1.53 | Fixed | 14 | Moderate | NS |
| J. Bai | 2018 | FHT | MD | -0.85 | -0.79 | 0.08 | Random | 96 | Low | Ⅳ |
| J. Bai | 2018 | VAS | MD | -0.59 | -1.18 | -0.01 | Random | 71 | Low | NS |
| J. Bai | 2018 | Malunion | RR | 0.34 | 0.06 | 2.05 | Fixed | 3 | Moderate | NS |
| J. Bai | 2018 | Infection | RR | 0.51 | 0.20 | 1.31 | Fixed | 0 | Moderate | NS |
| L. Xiong[9] | 2019 | OT | MD | -16.59 | -18.60 | -14.58 | Fixed | 23 | Moderate | NS |
| L. Xiong | 2019 | BL | SMD | -1.02 | -0.25 | -1.79 | Fixed | 20 | Moderate | NS |
| L. Xiong | 2019 | NOF | SMD | -2.20 | -2.50 | -1.90 | Fixed | 0 | Moderate | NS |
| L. Xiong | 2019 | FHT | SMD | 0.09 | -0.29 | 0.47 | NA | 51 | Low | NS |
| L. Xiong | 2019 | REGO | OR | 1.17 | 0.59 | 2.33 | Fixed | 0 | Moderate | NS |
| L. Xiong | 2019 | FRR | OR | 2.29 | 0.55 | 9.45 | Fixed | 0 | Moderate | NS |
| L. Xiong | 2019 | PC | OR | 0.59 | 0.24 | 1.45 | Fixed | 0 | Moderate | NS |
| L. Xiong | 2019 | LOHS | MD | 2.51 | 0.31 | 4.72 | Random | 81 | Low | Ⅳ |
| D. Zhu[10] | 2020 | OT | MD | -14.52 | -21.79 | -7.24 | Random | 97 | Low | Ⅳ |
| D. Zhu | 2020 | NOF | MD | -2.14 | -3.43 | -0.85 | Random | 95 | Low | Ⅳ |
| D. Zhu | 2020 | BL | MD | -13.59 | -18.07 | -9.10 | Random | 74 | Low | Ⅳ |
| D. Zhu | 2020 | VAS | MD | -0.55 | -1.72 | 0.62 | Random | 91 | Low | Ⅳ |
| D. Zhu | 2020 | G–W | MD | 0.56 | -4.18 | 5.30 | Fixed | 0 | Moderate | NS |
| D. Zhu | 2020 | REGO | RR | 1.10 | 0.96 | 1.27 | Fixed | 0 | Moderate | NS |
| J. Cao[11] | 2021 | OT | WMD | -38.84 | -54.89 | -22.78 | Random | 14.5 | Moderate | NS |
| J. Cao | 2021 | BL | WMD | -259.74 | –394.63 | –124.85 | Random | 30.1 | Moderate | NS |
| J. Cao | 2021 | FRR | RR | 0.56 | 0.38 | 0.81 | Random | 0 | Moderate | NS |
| J. Cao | 2021 | FHT | WMD | 34.07 | –49.02 | 19.12 | Random | 21.4 | Moderate | NS |
| J. Cao | 2021 | FOHJ | RR | 0.53 | 0.34 | 0.82 | Random | 0 | Moderate | NS |
| J. Cao | 2021 | PC | RR | 1.19 | 1.07 | 1.33 | Random | 0 | Moderate | NS |
| A. K. X. Lee[12] | 2022 | OT | ROM | 0.74 | 0.66 | 0.83 | Random | 93 | Moderate | Ⅳ |
| A. K. X. Lee | 2022 | BL | ROM | 0.71 | 0.63 | 0.81 | Random | 71 | Moderate | Ⅳ |
| A. K. X. Lee | 2022 | NOF | ROM | 0.36 | 0.17 | 0.76 | Random | 99 | Moderate | Ⅳ |
| A. K. X. Lee | 2022 | PC | OR | 0.42 | 0.22 | 0.78 | Random | 9 | High | NS |
| A. K. X. Lee | 2022 | FRR | OR | 1.53 | 1.08 | 2.17 | Random | 0 | High | NS |
| L. Xie[13] | 2018 | OT | SMD | -2.33 | -2.57 | -2.09 | Random | 91.3 | Low | Ⅳ |
| L. Xie | 2018 | BL | SMD | -1.51 | -1.72 | -1.29 | Random | 88.1 | Low | Ⅳ |
| L. Xie | 2018 | FHT | SMD | -1.90 | -2.24 | -1.56 | Random | 65.7 | Low | Ⅳ |
| L. Xie | 2018 | FFO | RR | 1.20 | 1.09 | 1.32 | Fixed | 0 | Moderate | NS |
| L. Xie | 2018 | PC | RR | 1.02 | 0.97 | 1.08 | Fixed | 0 | Moderate | NS |
| D. P. Tu[14] | 2021 | OT | SMD | -1.19 | -1.55 | -0.82 | Random | 66.1 | Moderate | Ⅳ |
| D. P. Tu | 2021 | BL | SMD | -1.08 | -1.65 | -0.51 | Random | 84.6 | Moderate | Ⅳ |
| D. P. Tu | 2021 | NOF | SMD | -1.64 | -2.35 | -0.93 | Random | 86.3 | Moderate | Ⅳ |
| D. P. Tu | 2021 | PC | OR | 0.43 | 0.24 | 0.79 | Fixed | 24 | High | NS |
| D. P. Tu | 2021 | MS | OR | 0.60 | 0.34 | 1.06 | Fixed | 0 | High | NS |

LOH: Loss of height; PFS: Postoperative functional scores; LOHS: Length of hospital stay; FOHJ: function of the hip joint; FFO: Follow-up functional outcomes; RS: Rasmussen scores; HSS: HSS scores; G–W: G–W scores; MS: Matta score; CS: Constant scores; OT: Operation time; BL: Blood loss; PC: Postoperative complications; REGO: the rate of excellent and good outcome; FHT: Fracture healing time; NOF: Number of fluoroscopies; FRR: fracture reduction rate; NA: Not access; NS: Not significant

Reference：

1. Shi, G., et al., *3D printing-assisted extended lateral approach for displaced intra-articular calcaneal fractures: a systematic review and meta-analysis.* Journal of orthopaedic surgery and research, 2021. **16**(1): p. 682.

2. Li, K., et al., *3D printing-assisted surgery for proximal humerus fractures: a systematic review and meta-analysis.* European journal of trauma and emergency surgery : official publication of the European Trauma Society, 2022. **48**(5): p. 3493-3503.

3. González-Alonso, M., et al., *Application of 3D printing in the treatment of appendicular skeleton fractures: Systematic review and meta-analysis.* Journal of Orthopaedic Research, 2021. **39**(10): p. 2083-2092.

4. He, Y., P. Zhou, and C. He, *Clinical efficacy and safety of surgery combined with 3D printing for tibial plateau fractures: systematic review and meta-analysis.* Annals of Translational Medicine, 2022. **10**(7).

5. Yammine, K., et al., *Clinical outcomes of the use of 3D printing models in fracture management: a meta-analysis of randomized studies.* European journal of trauma and emergency surgery : official publication of the European Trauma Society, 2022. **48**(5): p. 3479-3491.

6. Wang, J., et al., *Comparison of the feasibility of 3D printing technology in the treatment of pelvic fractures: a systematic review and meta-analysis of randomized controlled trials and prospective comparative studies.* European journal of trauma and emergency surgery : official publication of the European Trauma Society, 2021. **47**(6): p. 1699-1712.

7. Wood, L. and Z. Ahmed, *Does using 3D printed models for pre-operative planning improve surgical outcomes of foot and ankle fracture fixation? A systematic review and meta-analysis.* European journal of trauma and emergency surgery : official publication of the European Trauma Society, 2024. **50**(1): p. 21-35.

8. Bai, J., et al., *Efficacy and safety of 3D print-assisted surgery for the treatment of pilon fractures: a meta-analysis of randomized controlled trials.* Journal of orthopaedic surgery and research, 2018. **13**(1): p. 283.

9. Xiong, L., et al., *The efficacy of 3D printing-assisted surgery for traumatic fracture: A meta-analysis.* Postgraduate Medical Journal, 2019. **95**(1126): p. 414-419.

10. Zhu, D., et al., *The efficacy of 3D printing-assisted surgery in treating distal radius fractures: Systematic review and meta-analysis.* Journal of Comparative Effectiveness Research, 2020. **9**(13): p. 919-931.

11. Cao, J., H. Zhu, and C. Gao, *A Systematic Review and Meta-Analysis of 3D Printing Technology for the Treatment of Acetabular Fractures.* BioMed Research International, 2021. **2021**.

12. Lee, A.K.X., et al., *Three-Dimensional Printing and Fracture Mapping in Pelvic and Acetabular Fractures: A Systematic Review and Meta-Analysis.* Journal of Clinical Medicine, 2022. **11**(18).

13. Xie, L., et al., *Three-dimensional printing assisted ORIF versus conventional ORIF for tibial plateau fractures: A systematic review and meta-analysis.* International Journal of Surgery, 2018. **57**: p. 35-44.

14. Tu, D.P., et al., *Three-dimensional printing combined with open reduction and internal fixation versus open reduction and internal fixation in the treatment of acetabular fractures: A systematic review and meta-analysis.* Chinese Journal of Traumatology - English Edition, 2021. **24**(3): p. 159-168.
